# A simple score to predict severe leptospirosis

**DOI:** 10.1371/journal.pntd.0007205

**Published:** 2019-02-13

**Authors:** Simon Smith, Brendan J. Kennedy, Alexis Dermedgoglou, Suzanne S. Poulgrain, Matthew P. Paavola, Tarryn L. Minto, Michael Luc, Yu-Hsuan Liu, Josh Hanson

**Affiliations:** 1 Department of Medicine, Cairns Hospital, Cairns, Queensland, Australia; 2 James Cook University, Cairns Campus, Cairns, Queensland, Australia; 3 Infectious Diseases Service, Royal Adelaide Hospital, Adelaide, South Australia, Australia; 4 Communicable Disease Control Branch, Adelaide, South Australia, Australia; 5 Department of Intensive Care, Cairns Hospital, Cairns, Queensland, Australia; 6 The Kirby Institute, University of New South Wales, Sydney, Australia; University of Connecticut Health Center, UNITED STATES

## Abstract

**Background:**

The case-fatality rate of severe leptospirosis can exceed 50%. While prompt supportive care can improve survival, predicting those at risk of developing severe disease is challenging, particularly in settings with limited diagnostic support.

**Methodology/Principal findings:**

We retrospectively identified all adults with laboratory-confirmed leptospirosis in Far North Queensland, Australia, between January 1998 and May 2016. Clinical, laboratory and radiological findings at presentation were correlated with the patients’ subsequent clinical course. Medical records were available in 402 patients; 50 (12%) had severe disease. The presence of oliguria (urine output ≤500 mL/24 hours, odds ratio (OR): 16.4, 95% confidence interval (CI): 6.9–38.8, p<0.001), abnormal auscultatory findings on respiratory examination (OR 11.2 (95% CI: 4.7–26.5, p<0.001) and hypotension (systolic blood pressure ≤100 mmHg, OR 4.3 (95% CI 1.7–10.7, p = 0.002) at presentation independently predicted severe disease. A three-point score (the SPiRO score) was devised using these three clinical variables, with one point awarded for each. A score could be calculated in 392 (98%) patients; the likelihood of severe disease rose incrementally: 8/287 (3%), 14/70 (20%), 18/26 (69%) and 9/9 (100%) for a score of 0, 1, 2 and 3 respectively (p = 0.0001). A SPiRO score <1 had a negative predictive value for severe disease of 97% (95% CI: 95–99%).

**Conclusions/Significance:**

A simple, three-point clinical score can help clinicians rapidly identify patients at risk of developing severe leptospirosis, prompting early transfer to referral centres for advanced supportive care. This inexpensive, bedside assessment requires minimal training and may have significant utility in the resource-limited settings which bear the greatest burden of disease.

## Author summary

Leptospirosis, a neglected tropical disease with a global distribution, is estimated to kill 60,000 people every year. Predicting those at risk of developing severe disease is challenging, and a simple scoring system to quantify the risk of severe disease has proven elusive. Identifying the high-risk patient is important, as it might expedite the initiation of life-saving supportive care. This review of 402 adult patients with leptospirosis in tropical Australia determined that three clinical variables identified at presentation independently predicted severe disease (a subsequent requirement for Intensive Care Unit admission, intubation, vasopressor support, renal replacement therapy or the development of pulmonary haemorrhage). These three variables (abnormal auscultatory findings on respiratory examination, hypotension and oliguria) were used to generate a simple, three-point clinical score which can be determined rapidly and reliably at the bedside by health care workers with minimal training. This simple score may help the clinical management of patients with leptospirosis, particularly in lower and middle-income countries that bear the greatest burden of disease.

## Introduction

Leptospirosis is a zoonotic infection with a global distribution [1, 2]. Although most infections are mild and self-limiting, the disease is believed to kill almost 60,000 people every year [1]. Severe disease–manifesting as pulmonary haemorrhage, acute kidney injury (AKI) or multiorgan failure–develops in 5–15% of cases. The case-fatality rate of severe leptospirosis is as low as 6% if there is prompt access to vasopressors, renal replacement therapy (RRT) and mechanical ventilation [3], but it can rise to greater than 50% if the delivery of this supportive care is delayed [4].

However, identifying the patients who are at risk of developing severe disease can be difficult. Different studies have suggested that the presence of a variety of clinical features, laboratory investigations and imaging and electrocardiography findings can help [5–10]. While these approaches may be helpful in well-resourced settings where there is access to advanced laboratory and radiology support, they may have less utility in low and middle-income countries (LMIC), which bear a disproportionate burden of the disease [1].

Leptospirosis is endemic in tropical northern Australia, and the state of Queensland has one of the highest reported incidences in the developed world [11]. Most of the cases in Queensland occur in relatively remote locations where there is limited access to diagnostic support. Accordingly, given the potential for patient deterioration, if there is clinical uncertainty about a patient’s prognosis, they are often transferred–sometimes great distances–to a tertiary centre for continuing care. Not only is this frequently unnecessary, it is inconvenient for patients and their families, and expensive for the health system.

To improve the triage of patients with leptospirosis, and identify patient characteristics that predict severe disease, we reviewed the presentation of adults with confirmed leptospirosis in Far North Queensland and correlated their clinical findings and laboratory and imaging results with their subsequent clinical course. Our aim was to produce a simple score that could be used to quickly identify the patients at greatest risk of deterioration, expediting their referral for intensive care unit (ICU) support. We also hoped that the score could predict which patients could be safely managed without transfer, providing reassurance for local clinicians and reducing costs for the health system. Recognising that leptospirosis has a significant burden in LMIC–and in remote locations in high-income countries–it was also hoped that the score that might be applicable where access to diagnostic support is limited.

## Methods

### Study population and data collection

This retrospective study was performed at Cairns Hospital, a 531-bed, tertiary referral hospital in tropical, northern Australia that–with 16 smaller community hospitals–provides medical services to a population of approximately 280,000 people across an area of 380,000km^2^. The local electronic pathology reporting system (AUSLAB) was used to identify all leptospirosis cases in the region between January 1998 and May 2016.

Adult patients (≥16 years of age) were defined as having confirmed leptospirosis if they met one or more of the following criteria: (1) Leptospires isolated from blood culture; (2) Microscopic agglutination test (MAT) single titre of ≥ 1:400; (3) Fourfold rise in MAT antibody titres; (4) Detection of *Leptospira* in blood by polymerase chain reaction (PCR).

Medical charts were reviewed at the hospital of first presentation and at Cairns Hospital if a patient required inter-hospital transfer. It was recognized that a proportion of the medical records would be unavailable as the health service has a policy of destroying the paper medical record if there have been no new patient encounters for ten years.

Patient characteristics at the time of presentation to medical attention were reviewed. The World Health Organization was undertaking enhanced surveillance of leptospirosis in the region and a case report form was in use for much of the study period. Clinical findings, haematology, biochemistry, urinalysis, chest x-ray and electrocardiogram results were recorded. The following cut-offs–based on the literature, reference ranges and everyday clinical practice–were used to characterise any association severe disease: hypotension (systolic blood pressure ≤100 mmHg), anaemia (haemoglobin ≤100 g/L), severe thrombocytopenia (platelets ≤50 x 10^9^/L), acidosis (bicarbonate ≤22 mmol/L), AKI (creatinine ≥2 mg/dL), jaundice (bilirubin ≥3 mg/dL) and C-reactive protein ≥200 mg/L. The quick Sequential Organ Failure Assessment (qSOFA) and the quick National Early Warning Score (qNEWS) scores were calculated for the patients with sufficient clinical information [12, 13].

### Outcome

Severe disease was defined as the development of pulmonary haemorrhage, ICU admission, or a requirement for RRT, intubation or vasopressor support. Pulmonary haemorrhage was said to be present if there was frank haemoptysis or if blood was present on tracheal aspirate.

### Ethical considerations

The study obtained approval from the Far North Queensland Human Research Ethics Committee (HREC/16/QCH/37 – 1043LR). As per the approval, this retrospective study used anonymized patient data and did not obtain individual patient consent. This study reviewed human patients only; no animals were involved in any aspect of the study.

### Statistical analysis

Data were entered into an electronic database (Microsoft Excel) and analysed using statistical software (Stata 14.0). Groups were analysed using the Kruskal-Wallis and chi-squared tests. Multivariate analysis was performed using backwards linear and logistic regression. For the multivariate analysis, only variables with an area under the receiver operating characteristic (AUROC) curve of >0.7 in univariate analysis were selected.

## Results

**Fig 1:**
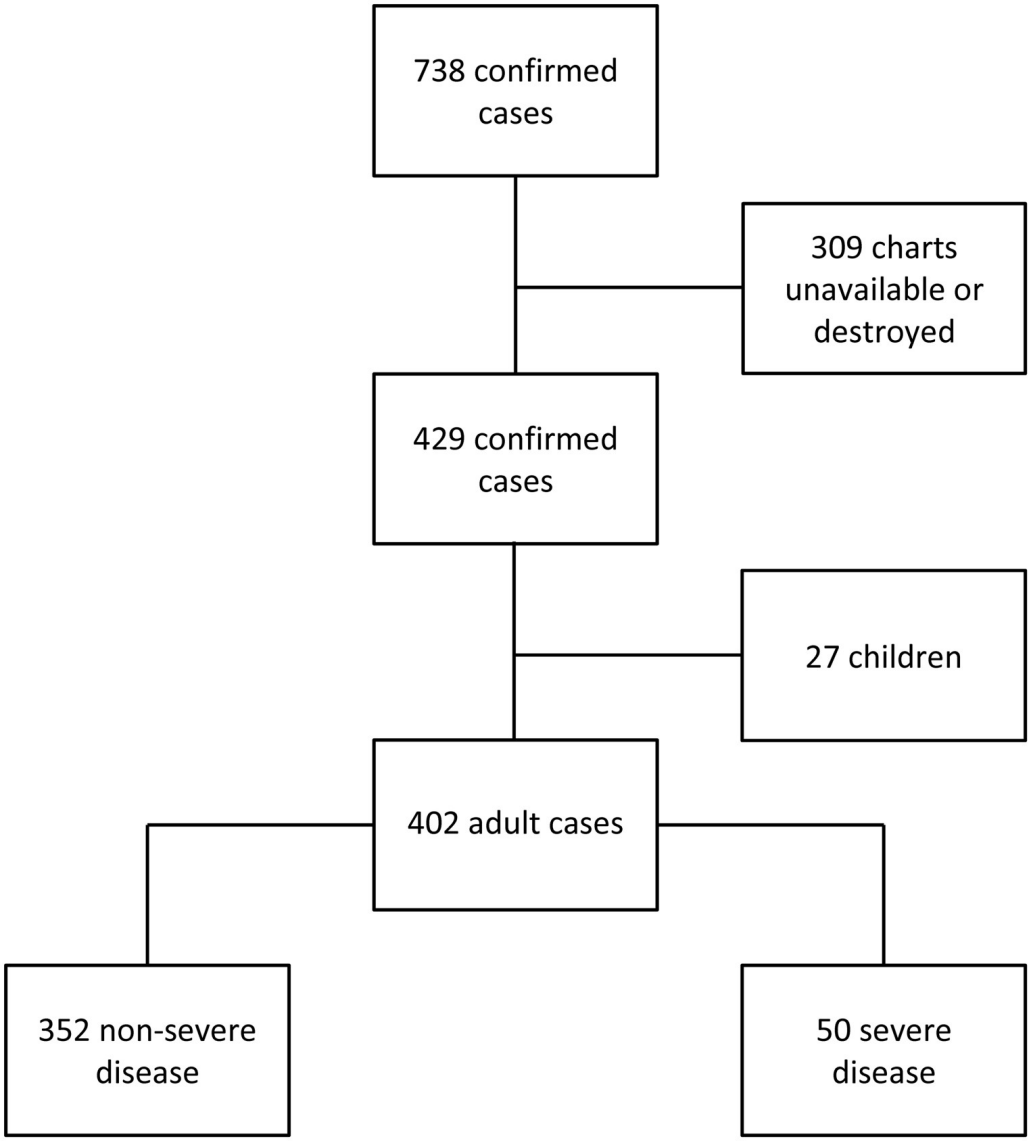
Consort diagram illustrating the number of confirmed cases of leptospirosis in Far North Queensland, Australia between 1998 and 2016.

There were 738 cases of laboratory-confirmed leptospirosis during the study period. Medical charts were available in 429 cases; 402 (94%) were adults. Their median (interquartile range (IQR)) age was 33 (23–45) years; 362 (90%) were male. Nearly all the cases (397/402 (99%)) were acquired locally, 273/397 (69%) occurred during the region’s November-April wet season, and 355/397 (89%) occurred in a region of high-intensity banana and dairy cattle farming situated approximately 100km south of Cairns. In the 384 in whom an occupation was documented, 327 (85%) had the potential for occupational exposure. There were 50 (12%) patients who developed severe disease, including two (0.5%) who died (Fig 1).

In 331/402 (82%) cases, clinicians included leptospirosis in the differential diagnosis at the time of presentation. Leptospires were isolated from blood culture in 275/402 (68%), MAT was diagnostic in 178/402 (44%) and PCR was positive in 151/402 (38%). Serovars could be determined in 353/402 (88%); Australis and Zanoni were the commonest serovars, and the most likely to cause severe disease (Table 1).

**Table 1:** Commonest serovars causing leptospirosis in Far North Queensland, Australia between 1998 and 2016.

Serovar	N	Non-severe disease	Severe disease
Zanoni	121	104 (86%)	17 (14%)
Australis	80	62 (78%)	18 (22%)
Arborea	57	57 (100%)	0 (0%)
Kremastos	25	23 (92%)	2 (8%)
Hardjo	20	17 (85%)	3 (15%)
Robinsoni	15	14 (93%)	1 (7%)
Szwajizak	12	12 (100%)	0 (0%)

The median (IQR) duration of symptoms was 4 (3–6) days in the patients who developed severe disease compared with 3 (2–4) days in those that did not (p = 0.0001). Comorbidities were documented in 395 patients and were more common in the patients who had severe disease (15/49 (31%) than those that did not (29/346 (8%), p<0.0001).

Clinical findings were similar to those in the published literature, although only 9% had a serum bilirubin ≥3mg/dL and conjunctival suffusion was documented in only 109/397 (27%) who were assessed. The symptoms, signs and laboratory tests and their association with severe disease are presented in Tables 2 and 3. In univariate analysis, the symptoms of diarrhoea, dyspnoea and bleeding were most associated with the development of severe disease (Table 2). The presence of renal impairment and thrombocytopenia were the laboratory tests most associated with the development of severe disease (Table 3).

**Table 2:** Presenting symptoms and signs of non-severe and severe leptospirosis.

	N	Non-severe disease n = 352	Severe disease n = 50	p	AUROC
Bleeding	397	7 (2%)	20 (41%)	<0.0001	0.69 (0.62–0.76)
Diarrhoea	398	79 (22%)	24 (49%)	<0.0001	0.63 (0.56–0.71)
Dyspnoea	397	14 (4%)	12 (24%)	<0.0001	0.60 (0.54–0.66)
Hepatomegaly	396	15 (4%)	10 (20%)	<0.0001	0.58 (0.52–0.64)
Abdominal pain	398	75 (21%)	17(35%)	0.04	0.57 (0.50–0.64)
Nausea/vomiting	398	238 (68%)	39 (80%)	0.10	0.56 (0.49–0.62)
Cough	397	97 (28%)	20 (41%)	0.06	0.56 (0.49–0.64)
Rash	397	19 (5%)	5 (10%)	0.19	0.52 (0.48–0.57)
Jaundice	396	9 (3%)	3 (6%)	0.18	0.52 (0.48–0.55)
Fevers/chills/sweats	397	337 (97%)	47 (96%)	0.74	0.50 (0.47–0.52)
Back pain	396	85 (25%)	11 (22%)	0.75	0.49 (0.43–0.55)
Arthralgia/myalgia	397	291 (84%)	39 (80%)	0.48	0.48 (0.42–0.54)
Conjunctival suffusion	397	98 (28%)	11 (22%)	0.40	0.47 (0.41–0.54)
Lymphadenopathy	397	25 (7%)	1 (2%)	0.17	0.47 (0.45–0.50)
Headache	397	291 (84%)	35 (71%)	0.037	0.44 (0.37–0.51)

**Table 3:** Features of leptospirosis at presentation and their association with subsequent severe disease.

Variable	N	Non-severe disease n = 352	Severe disease n = 50	p	AUROC
Age (years)	402	31 (23–42)	48 (34–61)	0.0001	0.74 (0.67–0.81)
Days of symptoms	390	3 (2–4)	4 (3–6)	0.0001	0.77 (0.71–0.84)
Abnormal respiratory auscultation	397	22 (6%)	28 (57%)	<0.0001	0.75 (0.68–0.85)
Oliguria	397	19 (5%)	29 (59%)	<0.0001	0.77 (0.70–0.84)
Systolic blood pressure (mmHg)	393	120 (110–130)	100 (90–116)	0.0001	0.73 (0.64–0.81)
Hemoglobin (g/L)	390	146 (138–154)	130 (117–147)	0.0001	0.73 (0.64–0.82)
WBC (x 10^9^/L)	387	8.7 (6.5–10.7)	10 (8.4–13.9)	0.0002	0.67 (0.59–0.75)
Neutrophils (x 10^9^/L)	387	7.5 (5.2–9.4)	8.9 (7.2–12.5)	0.0001	0.67 (0.59–0.75)
Platelets (x 10^9^/L)	370	154 (123–194)	97 (54–146)	0.0001	0.75 (0.66–0.83)
Sodium (mmol/L)	393	135 (132–136)	132 (128–135)	0.0001	0.67 (0.58–0.75)
Potassium (mmol/L)	378	3.7 (3.5–4.0)	3.6 (3.3–4.1)	0.12	0.57 (0.47–0.67)
Bicarbonate (mmol/L)	387	25 (23–26)	22 (20–25)	0.0001	0.71 (0.62–0.80)
Urea (mg/dL)	391	14.6 (11.8–18.5)	33.9 (20.4–60.8)	0.0001	0.85 (0.78–0.91)
Creatinine (mg/dL)	391	1.1 (0.9–1.3)	2.3 (1.4–4.8)	0.0001	0.86 (0.81–0.92)
Glucose (mg/dL)	207	117 (108–130)	123 (101–144)	0.15	0.58 (0.45–0.71)
Bilirubin (mg/dL)	386	0.9 (0.7–1.3)	1.2 (0.9–1.9)	0.0001	0.70 (0.63–0.78)
ALT (U/L)	385	32 (21–65)	46 (27–74)	0.01	0.61 (0.53–0.68)
LDH (U/L)	169	248 (206–317)	279 (214–376)	0.07	0.61 (0.49–0.73)
Creatine kinase (U/L)	43	130 (50–448)	505 (125–860)	0.03	0.70 (0.54–0.86)
Prothrombin time (s)	73	13 (11–14)	14 (12–15)	0.17	0.59 (0.46–0.73)
Lactate (mmol/L)	42	1.0 (0.8–2.0)	2.0 (1.1–5.8)	0.02	0.72 (0.56–0.88)
C-reactive protein	123	183 (100–257)	309 (233–335)	0.0001	0.78 (0.67–0.88)
CXR abnormal	154	16 (15%)	29 (62%)	<0.0001	0.73 (0.66–0.81)

Abbreviations: AUROC, Area under receiver operating curve; WBC, White blood cells; ALT, alanine aminotransferase; LDH, lactate dehydrogenase; CXR, Chest X-ray

In the 50 patients with severe disease, 45 (90%) required ICU admission, 27 (54%) developed pulmonary haemorrhage, 27 (54%) required vasopressor support, 18 (36%) required RRT and 24 (48%) required mechanical ventilation. APACHE III scores were available for 39/45 (87%) patients admitted to ICU; the median score was 84 (range 27–169).

There were only two deaths in the study. The first was an 80-year-old man with a history of diabetes mellitus and 5 days of symptoms; he developed multiorgan failure and died one day after presentation despite ICU support. The second, a 73-year-old man with a history of cardiovascular disease, chronic lung disease and connective tissue disease, had four days of symptoms; he also had multiorgan failure and died within one day of presentation.

**Fig 2:**
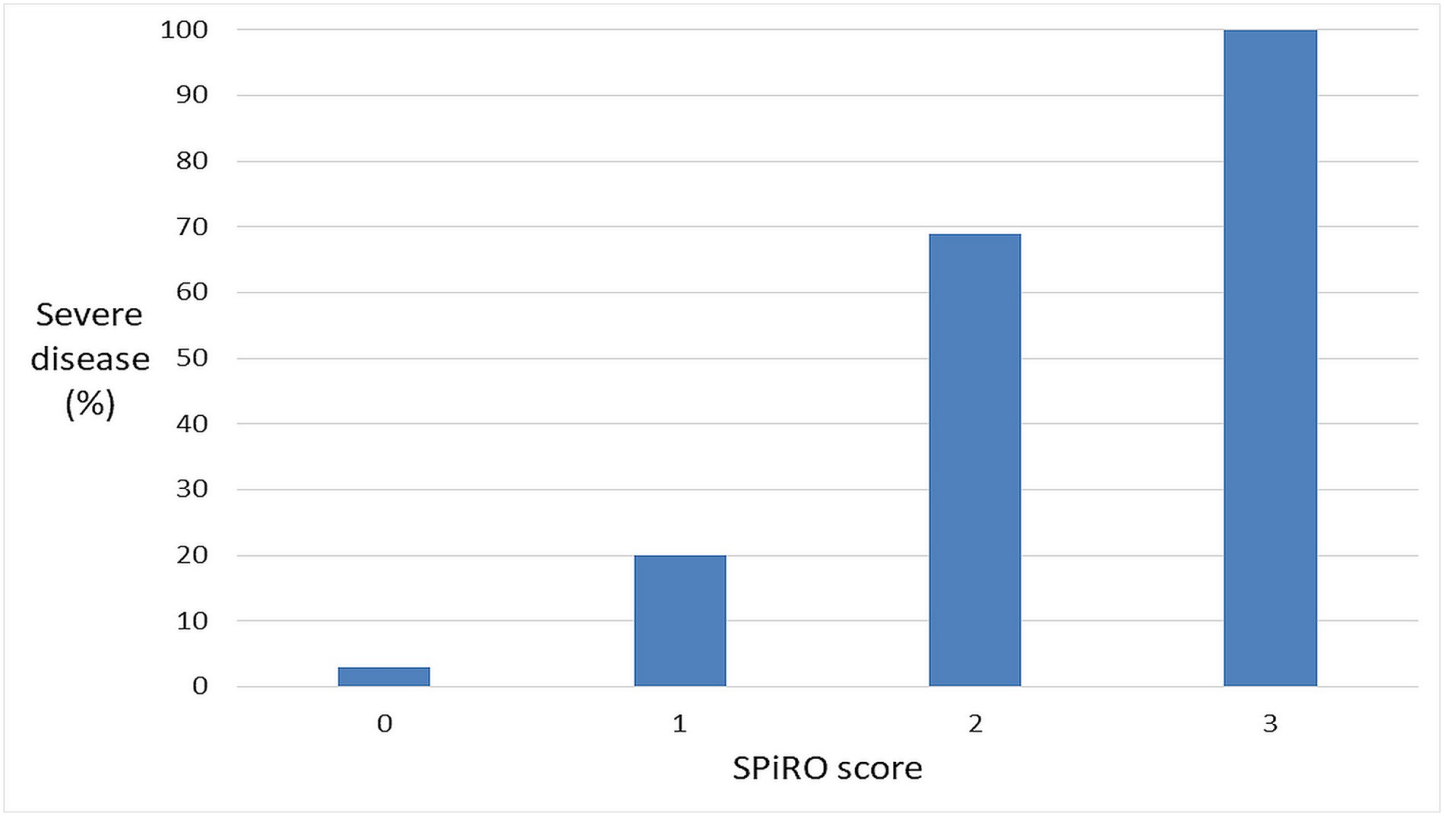
Positive predictive value of developing severe leptospirosis using the three-point SPiRO score calculated at presentation.

Multivariate analysis identified four independent variables associated with severe disease; three of these variables were clinical signs–abnormal auscultatory findings on respiratory examination (odds ratio (OR) (95% CI): 8.9 (3.5–22.4), p<0.0001), oliguria (OR (95% CI): 8.2 (3.2–21.2) p<0.0001) and hypotension (OR (95% CI): 3.8 (1.5–9.9), p = 0.006) and one was a laboratory variable (creatinine ≥2 mg/dL) (OR (95% CI): 7.0 (2.7–18.1) p<0.0001). The three clinical findings–awarded one point each–were used to generate a three-point SPiRO score (**S**ystolic blood **P**ressure ≤100 mmHg, **R**espiratory auscultation abnormalities, **O**liguria, Table 4). The risk of severe disease increased incrementally with the SPiRO score. A score of zero had a negative predictive value (NPV) for severe disease of 97.2% (95% CI: 94.6–98.8%). A score greater than one had a positive predictive value (PPV) (95% CI) for severe disease of 77.1% (59.9–89.6), while a score of three had a PPV of 100% (66.4–100) (Table 5 and Fig 2). The predictive ability of the SPiRO score was compared with the qSOFA and the qNEWS scores. In the 379/402 (94%) patients in whom the scores could be calculated, the AUROC of the SPiRO score (0.87 (95% CI 0.81–0.9) was higher than that of the qSOFA (0.76 (95% CI 0.70–0.83) score (p = 0.003). The difference between the AUROC of the SPiRO score and the qNEWS score (0.81 (95% CI 0.74–0.87) failed to reach statistical significance (p = 0.053).

**Table 4:** The SPiRO score for predicting severe leptospirosis.

Clinical feature	
**S**ystolic blood **P**ressure ≤100 mmHg	1 point
**R**espiratory auscultation abnormalities	1 point
**O**liguria	1 point

**Table 5:** Ability of the SPiRO score at presentation to predict severe leptospirosis at different cut-offs.

	0	1	2	3
Number of with severe disease/number of cases (%)	8/287 (3%)	14/70 (20%)	18/26 (69%)	9/9 (100%)
Sensitivity % (95% CI)	-	84% (70–93)	55% (40–69)	18% (9–32)
Specificity % (95% CI)	-	81% (77–85)	98% (96–99)	100% (99–100)
Positive predictive value % (95% CI)	-	39% (30–49)	77% (60–90)	100% (66–100)
Negative predictive value % (95% CI)	-	97% (95–99)	94% (91–96)	90% (86–92)

## Discussion

In adults with leptospirosis, a simple three-point clinical score–the SPiRO score–appears to reliably identify patients at risk of severe disease. The score could be used anywhere that leptospirosis is seen, but as a rapid and inexpensive assessment, which can be performed at the bedside by even junior health care workers, it has significant appeal for a disease that has its greatest impact in resource-poor settings. An absence of hypotension, oliguria or abnormal auscultatory findings–a SPiRO score of zero–was particularly helpful in identifying low-risk patients. The score could therefore determine which patients can be safely managed in remote locations, avoiding unnecessary and expensive transfer. The likelihood of severe disease rose incrementally as the score increased, facilitating recognition of the high-risk patient, expediting the initiation of supportive treatment and prompting consideration of transfer to referral centres.

Although many variables have been shown to predict severe leptospirosis, a simple scoring system to quantify the relative risk of severe disease has proven elusive [5]. As in this series, older age has been associated with severe leptospirosis and worse outcomes in multiple countries including India [4], Brazil [14] and Turkey [15]. Other predictors of severe leptospirosis or leptospirosis-attributable mortality have consisted of a combination of clinical features, laboratory findings and interpretation of imaging and electrocardiography. It is notable that pulmonary involvement is associated with worse outcomes in almost every published series, while renal involvement and hypotension also have also been shown to have significant prognostic utility.

In the French West Indies, dyspnoea, oliguria, white blood cell count >12,900/mm^3^, alveolar infiltrates on chest X-rays and repolarization abnormalities on electrocardiograms were independently associated with death [10]. In Brazil, pulmonary involvement, oliguria, creatinine >3 mg/dL and platelets <70,000/mm^3^ were independent predictors of mortality, with pulmonary involvement being the strongest prognostic factor [16]. Similarly, in India [17, 18], Indonesia [19], and Greece [20], pulmonary involvement was associated with increased mortality. In Thailand, pulmonary rales, oliguria, hypotension and hyperkalaemia were all independently associated with death [21].

While an elevated serum creatinine, white cell count and thrombocytopenia were also associated with severe disease in our series, it is important to remember that laboratory support may be limited in the rural and remote settings of LMIC where most cases of leptospirosis are seen. Even where there is access to laboratory support in these locations, results are not always available promptly. Similarly, while an abnormal chest X-ray had prognostic utility in our series, there may not be access to radiology support in leptospirosis-endemic areas and even when there is, accurate interpretation of imaging findings requires high quality images and significant medical training. Finally, although electrocardiography is inexpensive and relatively easy to perform, the identification of repolarization abnormalities also requires some expertise.

These issues may also be relevant in high-income settings like Australia. In rural locations where most cases of leptospirosis are diagnosed, it can take up to 24 hours for the processing, transport and analysis of even simple haematology and biochemistry tests such as platelet count or serum creatinine. Other laboratory tests that have been linked to severe leptospirosis, such as the quantification of leptospires in blood, are unlikely to be routinely available in the foreseeable future [6, 22, 23]. Imaging is also not necessarily accessible, and patients are usually reviewed initially by junior staff.

The entirely clinical SPiRO score therefore has significant appeal. It is simple to perform, reproducible, requires little medical training and addresses the renal impairment, pulmonary involvement and hypotension that have repeatedly been shown to be associated with the worst clinical outcomes [10, 16–21]. AKI in leptospirosis occurs due to direct leptospire invasion resulting in tubulointerstitial nephritis [24]. Renal biopsy most frequently reveals a mononuclear cellular infiltration and interstitial oedema, although an immune-complex glomerulonephritis may also be present [25, 26]. While leptospirosis has traditionally been thought to cause non-oliguric AKI [27], oliguria is an early clinical marker of AKI that is less likely to respond to rehydration and more likely to require RRT [28]. Hypotension in leptospirosis is usually due to vasodilatory mediators and proinflammatory cytokines released in response to the infection; this results in reduced renal blood flow, further exacerbating renal injury [29]. Pulmonary involvement–perhaps the most serious manifestation of severe disease–is frequently overlooked [23, 30]. Leptospirosis impairs the fluid handling of alveolar epithelial cells resulting in pulmonary oedema which can trigger acute respiratory distress syndrome [31–33]. Pulmonary haemorrhage–the most feared respiratory manifestation–is thought to occur from a direct effect of leptospiral proteins or toxic cellular components on multiple components of the alveolocapillary membrane [34]. As larger areas of haemorrhage coalesce, symptoms worsen and clinical signs are more likely to be apparent on auscultation [35]. As pulmonary haemorrhage progresses, pulmonary vascular resistance also increases, further contributing to systemic hypotension [29].

Severe disease was common in our series, but the case-fatality rate was very low with both deaths occurring in elderly patients with significant comorbidities. The wide variation in case-fatality rates reported in the literature has been attributed to differing definitions of severe leptospirosis, although our definition was conservative and the patients’ APACHE III scores were high. It is possible that the local case-fatality rate is higher than we have reported as patients with rapidly fatal leptospirosis may have a negative MAT test early in their disease. However, culture and PCR are used widely locally with only 18% of cases were diagnosed by MAT alone and accordingly, the number of unrecognized, fatal cases is probably small. The excellent outcomes are likely to be the result of early disease recognition and access to prompt ICU support. There is an extensive medical retrieval network in Australia which ensures people living in rural and remote services have access to sophisticated healthcare. However, given the country’s great expanse, these services are costly, frequently relying on a combination of road ambulances, helicopters and aeroplanes. While the coordination of retrieval services is centralised and efficient, organising safe and appropriate medical evacuation is time-consuming. A simple scoring system that facilitates recognition of patients with severe leptospirosis could help guide clinicians to identify which patients are most likely to require critical care support and early referral to retrieval services. Conversely, the SPiRO score could help prevent unnecessary medical evacuation, which would be welcome for patients and reassuring for the clinicians involved in their care.

Our study was retrospective and the SPiRO score requires prospective validation to ensure its applicability in other geographical settings. However, in other locations including Brazil [14, 36], Thailand [21], Moldova [37], Greece [20], and Réunion [38], hypotension, oliguria or abnormal respiratory auscultation have been identified previously as independent predictors of severe disease. Indeed, in a much smaller series from French Polynesia the same three clinical parameters were found to be the only independent variables in predicting severe disease [39]. The SPiRO score therefore has potential global utility. While many counties with a high incidence of leptospirosis do not have access to medical retrieval services or advanced ICU support, the score may still identify those who may benefit from closer monitoring and might be expected to improve outcomes.

The clinical findings of leptospirosis have been linked to the infecting serovar, with serogroup Icterohaemorrhagiae particularly associated with severe disease [6, 35]. This serogroup was uncommon in our series, occurring in only two cases, while severe disease was most commonly associated with serovars Australis and Zanoni. Variations in the prevalence of different serogroups and serovars have the potential to limit the generalizability of our findings, however, as previously noted, the clinical phenotype seen in our cohort was remarkably similar to that seen in the published literature [3, 10, 35].

Evidently, the SPiRO score can only be applied to patients with a diagnosis of leptospirosis, a condition whose prompt diagnosis remains challenging. While clinical findings can inform the clinician, they are non-specific and may not differentiate leptospirosis from other tropical infections including rickettsial disease, malaria and dengue. PCR is rarely available where the disease is endemic, and even in well-resourced settings like Australia, results take several days. Point-of-care tests have the greatest potential to facilitate diagnosis in both resource-poor and rich countries, but although their sensitivity and specificity is improving, these tests are not currently in routine use [40, 41]. If reliable point-of care tests can be developed and coupled with a validated simple predictive tool, the early recognition and management of leptospirosis is likely to improve significantly. That being said, clinicians in leptospirosis-endemic areas often recognize the disease–in our series, leptospirosis was in the initial differential diagnosis in over 82% of cases. Furthermore, even when the diagnosis of leptospirosis cannot be confirmed, a patient presenting to a remote clinic with hypotension and evidence of pulmonary and renal disease is likely to require referral for more sophisticated care whatever the aetiology.

The retrospective nature of our study meant that documentation was sometimes incomplete, and investigations were not standardised. However, clinicians working in the area have a high index of suspicion for the disease and a leptospirosis *pro forma* was in use for most of the study period. As a result, the clinical features on presentation were generally well documented. Data are now being collected prospectively to confirm these preliminary observations. The antibiotic therapy, its timing, route and duration were available in most cases and almost all patients received at least one appropriate agent. However, the enormous variety of antibiotic regimens prescribed precluded meaningful analysis of their relative efficacies.

In conclusion, a simple three-point clinical based scoring tool appears to help clinicians identify people at risk of developing severe leptospirosis. The score requires prospective validation in other geographical locations, but it has the potential to improve the care of people with leptospirosis, particularly in resource-limited settings where the disease has its greatest clinical burden.

## Supporting information

S1 Checklist STROBE checklist. (PDF)

## References

[1] Costa F, Hagan JE, Calcagno J, Kane M, Torgerson P, Martinez-Silveira MS, et al Global Morbidity and Mortality of Leptospirosis: A Systematic Review. PLoS neglected tropical diseases. 2015;9(9):e0003898 Epub 2015/09/18. 10.1371/journal.pntd.0003898 . 26379143 PMC4574773

[2] Hartskeerl RA, Collares-Pereira M, Ellis WA. Emergence, control and re-emerging leptospirosis: dynamics of infection in the changing world. Clinical microbiology and infection: the official publication of the European Society of Clinical Microbiology and Infectious Diseases. 2011;17(4):494–501. Epub 2011/03/19. 10.1111/j.1469-0691.2011.03474.x . 21414083

[3] Delmas B, Jabot J, Chanareille P, Ferdynus C, Allyn J, Allou N, et al Leptospirosis in ICU: A Retrospective Study of 134 Consecutive Admissions. Critical care medicine. 2018;46(1):93–9. Epub 2017/11/09. 10.1097/CCM.0000000000002825 . 29116996

[4] Chawla V, Trivedi TH, Yeolekar ME. Epidemic of leptospirosis: an ICU experience. The Journal of the Association of Physicians of India. 2004;52:619–22. Epub 2005/04/26. . 15847354

[5] Rajapakse S, Rodrigo C, Haniffa R. Developing a clinically relevant classification to predict mortality in severe leptospirosis. Journal of emergencies, trauma, and shock. 2010;3(3):213–9. Epub 2010/10/12. 10.4103/0974-2700.66519 . 20930963 PMC2938484

[6] Tubiana S, Mikulski M, Becam J, Lacassin F, Lefevre P, Gourinat AC, et al Risk factors and predictors of severe leptospirosis in New Caledonia. PLoS neglected tropical diseases. 2013;7(1):e1991 Epub 2013/01/18. 10.1371/journal.pntd.0001991 . 23326614 PMC3542117

[7] Lee N, Kitashoji E, Koizumi N, Lacuesta TLV, Ribo MR, Dimaano EM, et al Building prognostic models for adverse outcomes in a prospective cohort of hospitalised patients with acute leptospirosis infection in the Philippines. Transactions of the Royal Society of Tropical Medicine and Hygiene. 2017;111(12):531–9. Epub 2018/03/09. 10.1093/trstmh/try015 . 29518223

[8] Marotto PC, Ko AI, Murta-Nascimento C, Seguro AC, Prado RR, Barbosa MC, et al Early identification of leptospirosis-associated pulmonary hemorrhage syndrome by use of a validated prediction model. The Journal of infection. 2010;60(3):218–23. Epub 2009/12/23. 10.1016/j.jinf.2009.12.005 . 20026189 PMC3921886

[9] Hochedez P, Theodose R, Olive C, Bourhy P, Hurtrel G, Vignier N, et al Factors Associated with Severe Leptospirosis, Martinique, 2010–2013. Emerging infectious diseases. 2015;21(12):2221–4. Epub 2015/11/20. 10.3201/eid2112.141099 . 26583702 PMC4672444

[10] Dupont H, Dupont-Perdrizet D, Perie JL, Zehner-Hansen S, Jarrige B, Daijardin JB. Leptospirosis: prognostic factors associated with mortality. Clinical infectious diseases an official publication of the Infectious Diseases Society of America. 1997;25(3):720–4. Epub 1997/10/06. . 9314467 10.1086/513767

[11] Lau C, Smythe L, Weinstein P. Leptospirosis: an emerging disease in travellers. Travel medicine and infectious disease. 2010;8(1):33–9. Epub 2010/03/02. 10.1016/j.tmaid.2009.12.002 . 20188303

[12] Seymour CW, Liu VX, Iwashyna TJ, Brunkhorst FM, Rea TD, Scherag A, et al Assessment of Clinical Criteria for Sepsis: For the Third International Consensus Definitions for Sepsis and Septic Shock (Sepsis-3). Jama. 2016;315(8):762–74. Epub 2016/02/24. 10.1001/jama.2016.0288 . 26903335 PMC5433435

[13] Redfern OC, Smith GB, Prytherch DR, Meredith P, Inada-Kim M, Schmidt PE. A Comparison of the Quick Sequential (Sepsis-Related) Organ Failure Assessment Score and the National Early Warning Score in Non-ICU Patients With/Without Infection. Critical care medicine. 2018;46(12):1923–33. Epub 2018/08/22. 10.1097/CCM.0000000000003359 . 30130262

[14] Ko AI, Galvao Reis M, Ribeiro Dourado CM, Johnson WD Jr., Riley LW. Urban epidemic of severe leptospirosis in Brazil. Salvador Leptospirosis Study Group. Lancet (London, England). 1999;354(9181):820–5. Epub 1999/09/15. . 10485724 10.1016/s0140-6736(99)80012-9

[15] Cetin BD, Harmankaya O, Hasman H, Gunduz A, Oktar M, Seber E. Acute renal failure: a common manifestation of leptospirosis. Renal failure. 2004;26(6):655–61. Epub 2004/12/17. . 15600257 10.1081/jdi-200037154

[16] Spichler AS, Vilaca PJ, Athanazio DA, Albuquerque JO, Buzzar M, Castro B, et al Predictors of lethality in severe leptospirosis in urban Brazil. The American journal of tropical medicine and hygiene. 2008;79(6):911–4. Epub 2008/12/05. . 19052303 PMC2640419

[17] Bharadwaj R, Bal AM, Joshi SA, Kagal A, Pol SS, Garad G, et al An urban outbreak of leptospirosis in Mumbai, India. Japanese journal of infectious diseases. 2002;55(6):194–6. Epub 2003/02/28. . 12606828

[18] Clerke AM, Leuva AC, Joshi C, Trivedi SV. Clinical profile of leptospirosis in South gujarat. Journal of postgraduate medicine. 2002;48(2):117–8. Epub 2002/09/07. . 12215693

[19] Budiono E, Sumardi, Riyanto BS, Hisyam B, Hartopo AB. Pulmonary involvement predicts mortality in severe leptospirosis patients. Acta medica Indonesiana. 2009;41(1):11–4. Epub 2009/03/05. . 19258674

[20] Papa A, Theoharidou D, Antoniadis A. Pulmonary involvement and leptospirosis, Greece. Emerging infectious diseases. 2009;15(5):834–5. Epub 2009/05/01. 10.3201/eid1505.080270 . 19402988 PMC2687020

[21] Panaphut T, Domrongkitchaiporn S, Thinkamrop B. Prognostic factors of death in leptospirosis: a prospective cohort study in Khon Kaen, Thailand. International journal of infectious diseases: IJID: official publication of the International Society for Infectious Diseases. 2002;6(1):52–9. Epub 2002/06/05. . 12044303 10.1016/s1201-9712(02)90137-2

[22] Truccolo J, Serais O, Merien F, Perolat P. Following the course of human leptospirosis: evidence of a critical threshold for the vital prognosis using a quantitative PCR assay. FEMS microbiology letters. 2001;204(2):317–21. Epub 2001/12/04. 10.1111/j.1574-6968.2001.tb10904.x . 11731142

[23] Segura ER, Ganoza CA, Campos K, Ricaldi JN, Torres S, Silva H, et al Clinical spectrum of pulmonary involvement in leptospirosis in a region of endemicity, with quantification of leptospiral burden. Clinical infectious diseases: an official publication of the Infectious Diseases Society of America. 2005;40(3):343–51. Epub 2005/01/26. 10.1086/427110 . 15668855 PMC2366057

[24] Yang CW. Leptospirosis renal disease: understanding the initiation by Toll-like receptors. Kidney international. 2007;72(8):918–25. Epub 2007/08/10. 10.1038/sj.ki.5002393 . 17687261

[25] Lai KN, Aarons I, Woodroffe AJ, Clarkson AR. Renal lesions in leptospirosis. Australian and New Zealand journal of medicine. 1982;12(4):276–9. Epub 1982/08/01. . 6958239 10.1111/j.1445-5994.1982.tb03811.x

[26] Ooi BS, Chen BT, Tan KK, Khoo OT. Human renal leptospirosis. The American journal of tropical medicine and hygiene. 1972;21(3):336–41. Epub 1972/05/01. . 5025618 10.4269/ajtmh.1972.21.336

[27] Seguro AC, Lomar AV, Rocha AS. Acute renal failure of leptospirosis: nonoliguric and hypokalemic forms. Nephron. 1990;55(2):146–51. Epub 1990/01/01. 10.1159/000185943 . 2362627

[28] Leedahl DD, Frazee EN, Schramm GE, Dierkhising RA, Bergstralh EJ, Chawla LS, et al Derivation of urine output thresholds that identify a very high risk of AKI in patients with septic shock. Clinical journal of the American Society of Nephrology: CJASN. 2014;9(7):1168–74. Epub 2014/05/03. 10.2215/CJN.09360913 . 24789551 PMC4078959

[29] Sitprija V. Renal dysfunction in leptospirosis: a view from the tropics. Nature clinical practice Nephrology. 2006;2(12):658–9. Epub 2006/11/25. 10.1038/ncpneph0326 . 17124518

[30] Vinetz JM. Leptospirosis. Current opinion in infectious diseases. 2001;14(5):527–38. Epub 2002/04/20. . 11964872 10.1097/00001432-200110000-00005

[31] Dolhnikoff M, Mauad T, Bethlem EP, Carvalho CR. Leptospiral pneumonias. Current opinion in pulmonary medicine. 2007;13(3):230–5. Epub 2007/04/07. 10.1097/MCP.0b013e3280f9df74 . 17414132

[32] Andrade L, Rodrigues AC Jr., Sanches TR, Souza RB, Seguro AC. Leptospirosis leads to dysregulation of sodium transporters in the kidney and lung. American journal of physiology Renal physiology. 2007;292(2):F586–92. Epub 2006/08/31. 10.1152/ajprenal.00102.2006 . 16940563

[33] Sartori C, Matthay MA. Alveolar epithelial fluid transport in acute lung injury: new insights. The European respiratory journal. 2002;20(5):1299–313. Epub 2002/11/27. . 12449188 10.1183/09031936.02.00401602

[34] De Brito T, Aiello VD, da Silva LF, Goncalves da Silva AM, Ferreira da Silva WL, Castelli JB, et al Human hemorrhagic pulmonary leptospirosis: pathological findings and pathophysiological correlations. PloS one. 2013;8(8):e71743 Epub 2013/08/21. 10.1371/journal.pone.0071743 . 23951234 PMC3741125

[35] Levett PN. Leptospirosis. Clinical microbiology reviews. 2001;14(2):296–326. Epub 2001/04/09. 10.1128/CMR.14.2.296-326.2001 . 11292640 PMC88975

[36] Daher EF, Silva GB Jr., Karbage NN, Carvalho PC Jr., Kataoka RS, Silva EC, et al Predictors of oliguric acute kidney injury in leptospirosis. A retrospective study on 196 consecutive patients. Nephron Clinical practice. 2009;112(1):c25–30. Epub 2009/04/04. 10.1159/000210571 . 19342866

[37] Covic A, Goldsmith DJ, Gusbeth-Tatomir P, Seica A, Covic M. A retrospective 5-year study in Moldova of acute renal failure due to leptospirosis: 58 cases and a review of the literature. Nephrology, dialysis, transplantation: official publication of the European Dialysis and Transplant Association—European Renal Association. 2003;18(6):1128–34. Epub 2003/05/16. . 12748345 10.1093/ndt/gfg095

[38] Pertuiset E, Fen Chong M, Duval G, Genin R. [Clinical aspects and prognostic factors of icterohemorrhagic leptospirosis in adults. Apropos of 249 cases in La Reunion]. La Revue de medecine interne. 1988;9(5):487–93. Epub 1988/11/01. . 3067293 10.1016/s0248-8663(88)80012-2

[39] Doudier B, Garcia S, Quennee V, Jarno P, Brouqui P. Prognostic factors associated with severe leptospirosis. Clinical microbiology and infection: the official publication of the European Society of Clinical Microbiology and Infectious Diseases. 2006;12(4):299–300. Epub 2006/03/10. 10.1111/j.1469-0691.2005.01335.x . 16524404

[40] Nabity SA, Hagan JE, Araujo G, Damiao AO, Cruz JS, Nery N, et al Prospective evaluation of accuracy and clinical utility of the Dual Path Platform (DPP) assay for the point-of-care diagnosis of leptospirosis in hospitalized patients. PLoS neglected tropical diseases. 2018;12(2):e0006285 Epub 2018/02/21. 10.1371/journal.pntd.0006285 . 29462146 PMC5834199

[41] Goris MG, Leeflang MM, Loden M, Wagenaar JF, Klatser PR, Hartskeerl RA, et al Prospective evaluation of three rapid diagnostic tests for diagnosis of human leptospirosis. PLoS neglected tropical diseases. 2013;7(7):e2290 Epub 2013/07/23. 10.1371/journal.pntd.0002290 . 23875034 PMC3708816

